# Characterization of Plasmid-Mediated Quinolone Resistance (PMQR) Genes in Extended-Spectrum β-Lactamase-Producing *Enterobacteriaceae* Pediatric Clinical Isolates in Mexico

**DOI:** 10.1371/journal.pone.0077968

**Published:** 2013-10-17

**Authors:** Jesus Silva-Sánchez, Enrique Cruz-Trujillo, Humberto Barrios, Fernando Reyna-Flores, Alejandro Sánchez-Pérez, Bacterial Resistance Consortium, Ulises Garza-Ramos

**Affiliations:** 1 Instituto Nacional de Salud Pública, Centro de Investigación sobre Enfermedades Infecciosas (CISEI), Departamento de Diagnóstico Epidemiológico. Cuernavaca, Morelos, México; 2 Hospital Civil de Guadalajara “Fray Antonio Alcalde”, Instituto de Patología, Centro Universitario de Ciencias de la Salud, Universidad de Guadalajara. Guadalajara, Jalisco, México; University of Malaya, Malaysia

## Abstract

This work describes the characterization of plasmid-mediated quinolone-resistance (PMQR) genes from a multicenter study of ESBL-producing *Enterobacteriaceae* pediatric clinical isolates in Mexico. The PMQR gene-positive isolates were characterized with respect to ESBLs, and mutations in the GyrA and ParC proteins were determined. The phylogenetic relationship was established by PFGE and the transfer of PMQR genes was determined by mating assays. The prevalence of the PMQR genes was 32.1%, and the rate of qnr-positive isolates was 15.1%; 93.3% of the latter were *qnrB* and 6.4% were *qnrA1*. The distribution of isolates in terms of bacterial species was as follows: 23.5% (4/17) corresponded to *E. cloacae*, 13.7% (7/51) to *K. pneumoniae*, and 13.6% (6/44) to *E. coli*. In addition, the prevalence of *aac(6’)-Ib-cr* and *qepA* was 15.1% and 1.7%, respectively. The molecular characteristics of *qnr-* and *qepA-*positive isolates pointed to extended-spectrum β-lactamase (ESBL) CTX-M-15 as the most prevalent one (70.5%), and to SHV-12 in the case of *aac(6’)-Ib-cr-*positive isolates. GyrA mutations at codons Ser-83 and Asp-87, and ParC mutations at codons Ser-80 were observed in 41.1% and 35.2% of the *qnr-*positive isolates, respectively. The analysis of the transconjugants revealed a co-transmission of bla_CTX-M-15_ with the *qnrB* alleles. In general, the prevalence of PMQR genes (*qnr* and *aac(6’)-Ib-cr*) presented in this work was much lower in the pediatric isolates, in comparison to the adult isolates in Mexico. Also, ESBL CTX-M-15 was the main ESBL identified in the pediatric isolates, whereas in the adult ones, ESBLs corresponded to the CTX-M and the SHV families. In comparison with other studies, among the PMQR-genes identified in this study, the *qnrB*-alleles and the *aac(6’)-Ib-cr* gene were the most prevalent, whereas the *qnrS1*, *qnrA1* and *qnrB*-like alleles were the most prevalent in China and Uruguay.

## Introduction

 The use of β-lactam antibiotics for the treatment of bacterial infections caused by *Enterobacteriaceae* has been and will continue to be the main line of defense against these bacterial agents. However, bacterial resistance to these antibiotics has been increasing worldwide. In Latin America, Mexico is one of the countries with the highest consumption of antibiotics [1], and several studies in our country have confirmed the production of extended-spectrum β-lactamases (ESBLs) as the mechanism accounting for the β-lactam antibiotic resistance widely disseminated among *Enterobacteriaceae* [2,3]. The growing resistance to β-lactam antibiotics in the world has caused an increased prescription of quinolones and fluoroquinolones for the treatment of hospital- and community-acquired infections [4]. In contrast to the main mechanism of resistance to β-lactam antibiotics, quinolone and fluoroquinolone resistance arises by mutations in the chromosomal genes for type II topoisomerases, because they are the targets of quinolone action. However, other mechanisms has been described, such as changes in the expression of efflux pumps and porins that control the accumulation of the antibiotic agents inside the bacterial cell [5]. Nevertheless, transferable genes, which confer low resistance to quinolones and fluoroquinolones, have been identified recently, such as the *qnr* determinants whose genes encode for pentapeptide repeat proteins that bind to and protect type II DNA topoisomerases from inhibition by quinolones [6]. Since the first *qnr* determinant was described [7] and its subsequent distribution worldwide documented [8], this class of genes has been found to be larger, and multiple genes (*qnrA*-, *qnrB*-,*qnrS*-, *qnrC* and *qnrD*) have been characterized [9]. Moreover, other mechanisms have been identified, including the *aac(6*')*-Ib-cr* (modified acetyltransferase) and the *qepA* (efflux pump) genes [7,10]. Thus, these three resistance mechanisms are dependent on plasmid-mediated quinolone resistance (PMQR) genes. However, mutations in the chromosomal genes for type II topoisomerases are generally required for a clinically significant quinolone and fluoroquinolone resistance [11]. So far, the *qnr*-determinants are the most frequently identified ones [10]. Our group recently described PMQR genes in extended-spectrum β-lactamase (ESBL)-producing *Enterobacteriaceae* clinical isolates causing nosocomial infections in adults [12]; nevertheless, there are still no data regarding the pediatric population in Mexico. It is known, however, that quinolones should be prescribed with caution in pediatric hospitals, taking into account that arthropathy is their most worrisome side effect [13]. In addition, there are no routine indications for the use of quinolones for the pediatric population. In this study, we carried out the characterization of PMQR genes in ESBL-producing pediatric clinical isolates from a multicenter study in Mexico, and we identified the mutations in the *gyrA* and *parC* chromosomal genes as well as analyzed the transfer of quinolone and cephalosporin resistance genes.

## Materials and Methods

### Pediatric clinical isolates

For this study, 112 ESBL-producing *Enterobacteriaceae* pediatric clinical isolates were collected from five hospitals in four regions of Mexico: Hospital Civil de Guadalajara (HCG) in Jalisco, in the west; Hospital de Pediatría CMN Siglo XXI (HPCMN) in Mexico City, in the center; Hospital General de Tapachula Chiapas (HGTC) in Tapachula, Chiapas and Hospital del Niño (HN) in Villahermosa, Tabasco, in the south, and Hospital Universitario (HU) in Monterrey, Nuevo León, in the north. The isolates were analyzed at the National Institute of Public Health (Instituto Nacional de Salud Pública – INSP) in Cuernavaca, Morelos, México. Only one isolate per patient was used. 

Three ESBL-producing bacterial species--*Klebsiella pneumoniae* (51 isolates), *Escherichia coli* (44 isolates), and *Enterobacter cloacae* (17 isolates)--, isolated between 1996 and 2011 were included. ESBL production was detected according to guidelines of the Clinical and Laboratory Standards Institute (CLSI) (M100-S21) [14].

### Ethics Statement

This project was exempt from review by the Ethic Commission at INSP because it does not involve human subjects and/or it is not an academic study and/or it does not include the analysis of data previously obtained from another study requiring the patients’ informed consent. On the other hand, the bacteria included in the study were obtained by routine procedures in each of the hospitals involved.

### Screening of PMQR genes from pediatric isolates

 The quinolone resistance encoding-genes were screened for *qnr*-type genes (*qnrA*, *qnrB*, *qnrS, qnrC* and *qnrD*) in the 112 ESBL-producing *Enterobacteriaceae* pediatric isolates by means of separate multiplex-PCR methods [15], and confirmed by single PCR. The *qepA* and *aac(6*')*-Ib-cr* genes were analyzed by single PCR with specific primers for each gene [12]. The *aac(6*')*-Ib-cr* allele was differentiated from the wild-type gene by PCR digestion with *Bst*I5 enzyme and confirmed by nucleotide sequence. The *qepA* gene was amplified using a 1X enhancer buffer (Invitrogen, CA, USA). All PCR products were purified by means of a High Pure PCR Product Purification Kit (Roche Applied Science); they were sequenced using a chain termination method with a Big-Dye Terminator kit (Applied Biosystems Foster City, CA), and analyzed on an ABIPRISMA 3100 (Applied Biosystems). The nucleotide sequences were compared to the GenBank database by means of BLASTx searches.

### Analysis of PMQR gene-positive pediatric isolates

The CTX-M-, SHV-, and TLA-type ESBLs were screened by PCR using specific primers [12]. The MICs against nalidixic acid, ciprofloxacin, levofloxacin, cefotaxime, ceftazidime, ceftazidime/clavulanic acid and gentamicin were determined by the broth microdilution method following the recommendations of the Clinical and Laboratory Standards Institute (CLSI), and the results were interpreted according to CLSI performance standard M100-S21 [14]. *E. coli* ATCC 25922 was used as a reference strain for susceptibility testing. 

Both the *gyrA* and the *parC* chromosomal genes (with the exception of *E. cloacae parC*) were amplified by PCR with specific primers [12]. The quinolone resistance-determining regions (QRDRs) of the GyrA and the ParC proteins was identified, and the amino acid sequences were analyzed by BLASTx and compared to the protein sequences of GyrA and ParC proteins from *K. pneumoniae* and *E. coli* deposited in the GenBank. 

The transfer of the PMQR genes was explored by conjugation, according to method described by Miller [16]. Azide-resistant *E. coli* J53 was used as the recipient strain, and transconjugants were selected on LB plates containing sodium azide (100 mg/L), nalidixic acid (8 mg/L) or cefotaxime (1 mg/L). The phenotypic resistance was analyzed on LB agar plates with eight different types of antibiotics: nalidixic acid (8 mg/L), ciprofloxacin (0.5 mg/L), ampicillin (100 mg/L), cefotaxime (1 mg/L), tetracycline (15 mg/L), chloramphenicol (10 mg/L), kanamycin (25 mg/L), and gentamicin (16 mg/L). ESBLs and PMQR genes were identified as described above among the transconjugants. Plasmid incompatibility groups were analyzed in the isolates and their transconjugants by PCR-based replicon typing [17,18]. Plasmid DNA preparations were extracted from clinical isolates and transconjugants according to the method described by Kieser et al. [19]. Plasmids, 154-, 66-, 48- and 7-kb from *E. coli* NCTC 50192 [20] and pUA21 (300 kb) [12] were used as molecular size markers. The linear regression equation was used for molecular weight plasmid calculation.

All *qnr*-, *aac(6*’)*-Ib-c-r* and *qepA*-positive pediatric isolates were analyzed by pulsed-field gel electrophoresis (PFGE) [21], according to Kaufmann et al. [22]. The relationship between pediatric isolates was determined using the GelCompar II software (Applied Math, Kortrijk, Belgium). The similarity percentage was represented by means of a dendrogram derived from UPGMA and Dice coefficients (band position tolerance and optimization were set at 0.7% and 0.65%, respectively).

## Results

### Prevalence of PMQR genes among pediatric isolates

 The prevalence of PMQR genes (*qnr*, *aac(6*’)*-Ib-cr* and *qepA*) among *Enterobacteriaceae* pediatric isolates in Mexico was 32.1% (36/112). The prevalence of *qnr*-determinants was 15.1% (17/112), distributed as follows: 13.7% (7/51) among *K. pneumoniae* isolates, 13.6% (6/44) among *E. coli* isolates, and 23.5% (4/17) among *E. cloacae* isolates (Table 1). The prevalence with respect to bacterial genera was as follow: in *K. pneumoniae* isolates *qnr*-determinants was 13.7%, the *aac(6*’)*Ib-cr* gene was 33.3% and a 0% for *qepA*. In *E. coli* isolates *qnr*-determinants was 13.6%, the *aac(6*’)*Ib-cr* gene was 0% and a 4.5% for *qepA*. In *E. cloacae* isolates *qnr*-determinants was 23.5% and for *aac(6*’)*Ib-cr* and *qepA* genes was 0% (Table 1). Sixteen out of 17 (94.1%) isolates carried *qnrB* genes; type *qnrB1* turned out to be the most prevalent (47.0% - 8/16), followed by *qnrB5* (17.6% - 3/16), *qnrB20* and *qnrB19* (11.7% - 2/16), and *qnrB6* (5.8% - 1/16). The *qnrA1* gene was identified in 5.8% (1/17) of the isolates (Table 2). The *qnrS*, *qnrC* and *qnrD* genes were not detected. The prevalence of the *aac(6*')*-Ib-cr* and the *qepA1* genes was 15.1% (17/112) and 1.7% (2/112), and it corresponded to *K. pneumoniae* and *E. coli qnr*-negative isolates, respectively (Table 1).

**Table 1:** PMQR gene prevalence among ESBL-producing *Enterobacteriaceae* pediatric isolates.

Hospital (No. of isolates)							
Species	1. HCG	2. HPCMN	3. HGTC	4. HN	5. HU	Total	Prevalence
	(51)	(18)	(17)	(13)	(13)	(n = 112)	(%)
*qnr - aac(6’)-Ib-cr - qepA*							
*K. pneumoniae* (51)	2-0-0	0-0-0	0-17-0	3-0-0	2-0-0	7-17-0	13.7-33.3-0
*E. coli* (44)	1-0-0	0-0-2	0-0-0	0-0-0	5-0-0	6-0-2	13.6-0-4.5
*E. cloacae* (17)	2-0-0	0-0-0	0-0-0	0-0-0	2-0-0	4-0-0	23.5-0-0
Total	5-0-0	0-0-2	0-17-0	3-0-0	9-0-0	17-17-2	15.1-15.1-1.7

Abbreviations: 1. Hospital Civil de Guadalajara (HCG); 2. Hospital de Pediatría CMN Siglo XXI (HPCMN); 3. Hospital General de Tapachula Chiapas (HGTC); 4. Hospital del Niño de Tabasco (HN);

5 Hospital Universitario (HU).

**Table 2:** Molecular characteristics of PMQR gene-positive pediatric isolates.

Isolates	Species	Hospital^a^	Isolation date	PFGE^b^	Bacterial conjugation	Plasmid profile^c^	Plasmid Incompatibility group (Inc)	PMQR genes	GyrA^d^		ParC^d^	ESBL type			MIC	(g/L)			
									Ser83	Asp87	Ser80		NAL	CPO	LEV	CTX	CAZ	CAZ/CLV	Gm
4052	*E. cloacae*	1	23/08/2002	A (2)	-	100	ND	*qnrB20*	*-*	*-*	NA	ND	16	0.25	1	128	128	4	> 64
835	*E. cloacae*	5	14/03/2011	NR	+	320	FIIs	*qnrB1*	-	-	NA	ND^f^	16	0.5	1	> 256	>256	64	> 64
840	*E. cloacae*	5	09/04/2011	NR	+	240, 200, 130	FIIs, X	*qnrB19*	-	-	NA	CTX-M-15	8	0.0625	0.125	> 256	32	4	> 64
8019	*E. coli*	2	22/05/2009	B (2)	-	210, 150	ND	*qepA1*	Leu	Asn	Iso	CTX-M-15	> 256	> 64	> 64	> 256	64	4	4
836	*E. coli*	5	06/09/2010	C (2)	+	250, 160	FIIs, Frep, FIB	*qnrB1*	-	-	-	CTX-M-15	16	0.25	1	> 256	128	4	16
850	*E. coli*	5	01/12/2010	NR	+	200, 140, 60	FIIs	*qnrB19*	Leu	-	Iso	ND^f^	> 256	8	8	32	128	64	> 64
843	*E. coli*	5	03/09/2010	NR	+	260, 90	FIIs, Frep, FIB, L/M, HI1	*qnrB1*	-	-	-	CTX-M-15	32	0.25	1	> 256	128	4	4
844	*E. coli*	5	04/11/2010	NR	ND	250, 180	ND	*qnrB1*	-	-	-	CTX-M-15	8	0.25	4	> 256	64	4	16
01-1606	*E. coli*	1	24/02/2010	NR	-	260, 130, 110, 80, 60	ND	*qnrB6*	Leu	-	-	CTX-M-15	128	1	1	> 256	>256	128	8
06-1614	*K. pneumoniae*	5	14/04/2011	D (2)	ND	120, 90	ND	*qnrB5*	-	-	-	ND^g^	16	0.5	4	64	4	4	> 64
6737	*K. pneumoniae*	3	25/03/2007	E-NR (13-4^e^)	+	220, 60	FIIs	*aac(6’)Ib-cr*	-	-	-	SHV-12^f^,^g^	4	0.25	4	128	> 256	4	4
01-1600	*K. pneumoniae*	1	11/04/2010	NR	-	180	ND	*qnrA1*	Leu	-	Iso	CTX-M.15^g^	> 256	> 64	> 64	> 256	> 256	4	2
01-1634	*K. pneumoniae*	1	02/09/2010	NR	-	130, 80	ND	*qnrB1*	Iso	-	Iso	CTX-M-15^g^	> 256	> 64	> 64	> 256	128	4	2
06-1605	*K. pneumoniae*	4	17/02/2011	NR	+	180	FIIs	*qnrB5*	Leu	-	Iso	CTX-M-15^g^	> 256	> 64	16	> 256	> 256	4	> 64
839	*K. pneumoniae*	4	17/03/2011	NR	+	280	FIIs	*qnrB1*	-	-	-	ND^f^,^g^	32	1	4	> 256	> 256	32	> 64
837	*K. pneumoniae*	4	24/03/2011	NR	+	230	FIIs, FIB	*qnrB1*	-	-	-	CTX-M-15^g^	8	2	4	> 256	32	4	> 64

^a^ Hospitals: 1. Hospital Civil de Guadalajara (HCG); 2. Hospital de Pediatría CMN Siglo XXI (HPCMN); 3. Hospital General de Tapachula Chiapas (HGTC); 4. Hospital del Niño de Tabasco (HN); 5. Hospital Universitario (HU).

^b^ The XbaI restriction profiles showed different DNA patterns among the clinical isolates of their respective species. The number in parenthesis corresponds to the number of strains with the same PFGE pattern.

^c^ The underlined plasmids correspond to conjugative plasmids.

^d^ wild-type gene. Amino acids; Ser, serine; Asp, aspartic acid; Leu, leucine; Asn, asparagine.

^e^ In this hospital 17 pediatric isolates were analyzed; 13 corresponded to the same clone (E) and four were not related isolates.

^f^ β-lactamase TEM-1 was identified by PCR and sequencing.

^g^ β-lactamase SHV-11 was identified by PCR and sequencing.

NR, not related; NA, not analyzed; ND, not determined.

### Epidemiological Data and Characteristics of PMQR Gene-Positive Pediatric Isolates

 The age of the pediatric patients ranged from a few days to four years. Twenty-two out of 36 (61%) isolates corresponded to male patients, and 39% (14/36) to female patients. The samples came from the following sites: urine - 9 isolates (25%), secretion - 9 isolates (25%), catheter and LCR - 2 isolates (5.5% each), and blood - 16 isolates (44.5%).

**Figure 1:**
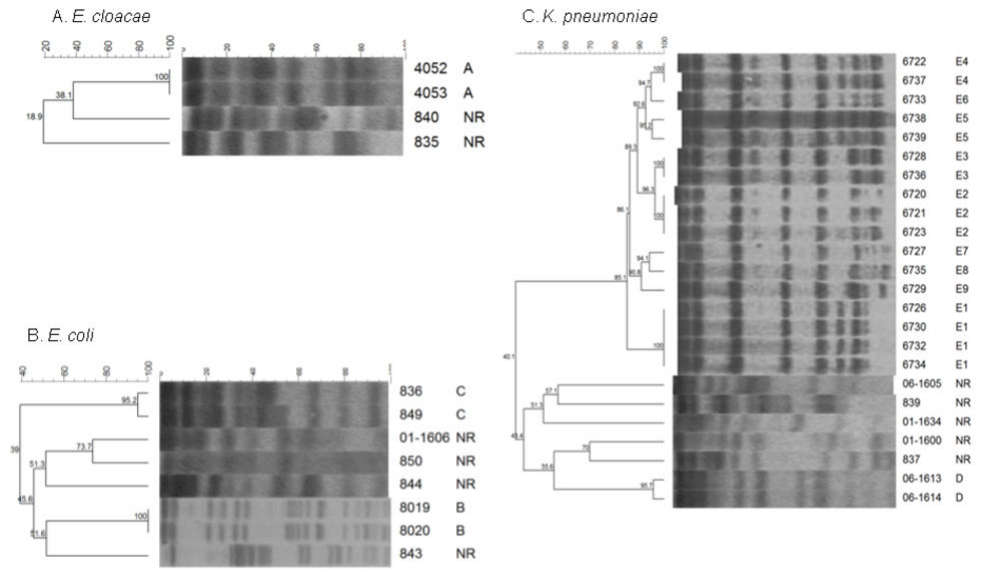
PFGE and dendrogram analysis of PMQR-positive *Enterobacteriaceae* pediatric isolates. A. *E. cloacae*, B. E. *coli* and C. *K. pneumoniae*.

The genotyping analysis of *qnr*-positive pediatric isolates showed a few genetic relationships between isolates in each group, and two isolates corresponded to each clone (A, B, C and D): clone A – *E. cloacae*; clones B and C – *E. coli*, and clone D - K*. pneumoniae*. However, 13 *aac(6*’)*-Ib-cr*-positive *K. pneumoniae* isolates corresponded to clone E (Table 2 and 3 and Figure 1). The *qnr*-positive isolates were subject to susceptibility testing that revealed the following: 41.1%, 29.4% and 52.9% were resistant to nalidixic acid, ciprofloxacin and levofloxacin, respectively. Four out of 17 *qnr*-positive isolates (23.5 %) showed MICs > 256 mg/L for nalidixic acid; three isolates (17.6%) showed MICs > 64 mg/L for ciprofloxacin, and three isolates (17.6%) showed MICs ranging from 16 to > 64 mg/L for levofloxacin. The remaining isolates were susceptible to nalidixic acid (58.8%), ciprofloxacin (70.5%) and levofloxacin (47%). With respect to gentamicin, 82.3% of the isolates were resistant to this antibiotic (mainly > 64 mg/L). All isolates showed resistance to cefotaxime, and 82.3% to ceftazidime (Table 2). These results in terms of cephalosporin antimicrobial susceptibility are consistent with the fact that ESBL CTX-M-15 was the most prevalent one (70.5% - 12/17). On the other hand, SHV- and TLA-1-type ESBLs were not detected, and it was not possible to identify the ESBL gene in 5 out of 17 isolates (Table 2).

**Table 3:** All PMQR gene-positive pediatric isolates.

Isolates	Species	Hospital^a^	Isolation date	PFGE^b^	Bacterial	Plasmid	PMQR	GyrA^d^		ParC^d^	ESBL type			MIC	(g/L)			
					conjugation	profile^c^	genes	Ser83	Asp87	Ser80		NAL	CPO	LEV	CTX	CAZ	CAZ/CLV	Gm
4052	*E. cloacae*	1	23/08/2002	A	+	100	*qnrB20*	*-*	*-*	NA	ND	16	0.25	1	128	128	4	> 64
4053	*E. cloacae*	1	24/08/2002	A	ND	100 ^f^	*qnrB20*	-	-	NA	ND	ND	ND	ND	ND	ND	ND	ND
835	*E. cloacae*	5	14/03/2011	NR	+	300	*qnrB1*	-	-	NA	ND^g^	16	0.5	1	> 256	>256	64	> 64
840	*E. cloacae*	5	09/04/2011	NR	+	240, 200, 130	*qnrB19*	-	-	NA	CTX-M-15	8	0.0625	0.125	> 256	32	4	> 64
8019	*E. coli*	2	22/05/2009	B	-	210, 150	*qepA1*	Leu	Asn	Iso	CTX-M-15	> 256	> 64	> 64	> 256	64	4	4
8020	*E. coli*	2	25/05/2009	B	ND	210^f^	*qepA1*	ND	ND	ND	CTX-M-15	ND	ND	ND	ND	ND	ND	ND
836	*E. coli*	5	06/09/2010	C	+	250, 160	*qnrB1*	-	-	-	CTX-M-15	16	0.25	1	> 256	128	4	16
849	*E. coli*	5	05/11/2010	C	ND	250, 160 ^f^	*qnrB1*	ND	ND	ND	CTX-M-15	ND	ND	ND	ND	ND	ND	ND
850	*E. coli*	5	01/12/2010	NR	+	200, 140, 60	*qnrB19*	Leu	-	Iso	ND^g^	> 256	8	8	32	128	64	> 64
843	*E. coli*	5	03/09/2010	NR	+	260, 90	*qnrB1*	-	-	-	CTX-M-15	32	0.25	1	> 256	128	4	4
844	*E. coli*	5	04/11/2010	NR	-	250, 180	*qnrB1*	-	-	-	CTX-M-15	8	0.25	4	> 256	64	4	16
01-1606	*E. coli*	1	24/02/2010	NR	+	260, 130, 110, 80, 60	*qnrB6*	Leu	-	-	CTX-M-15	128	1	1	> 256	>256	128	8
06-1614	*K. pneumoniae*	5	14/04/2011	D	-	120, 90	*qnrB5*	-	-	-	ND^h^	16	0.5	4	64	4	4	> 64
06-1613	*K. pneumoniae*	5	14/04/2011	D	ND	100, 80^f^	*qnrB5*	ND	ND	ND	ND	ND	ND	ND	ND	ND	ND	ND
6726	*K. pneumoniae*	3	15/03/2007	E1	+	220	*aac(6’)Ib-cr*	-	-	-	SHV-12^g^,^h^	4	0.25	4	256	> 256	8	4
6730	*K. pneumoniae*	3	21/03/2007	E1	ND	220, 60 ^f^	*aac(6’)Ib-cr*	ND	ND	ND	ND	ND	ND	ND	ND	ND	ND	ND
6732	*K. pneumoniae*	3	22/03/2007	E1	ND	220, 60 ^f^	*aac(6’)Ib-cr*	ND	ND	ND	ND	ND	ND	ND	ND	ND	ND	ND
6734	*K. pneumoniae*	3	23/03/2007	E1	ND	220 ^f^	*aac(6’)Ib-cr*	ND	ND	ND	ND	ND	ND	ND	ND	ND	ND	ND
6720	*K. pneumoniae*	3	08/08/2005	E2	ND	220, 60 ^f^	*aac(6’)Ib-cr*	ND	ND	ND	ND	ND	ND	ND	ND	ND	ND	ND
6721	*K. pneumoniae*	3	09/08/2005	E2	ND	220, 60 ^f^	*aac(6’)Ib-cr*	ND	ND	ND	ND	ND	ND	ND	ND	ND	ND	ND
6723	*K. pneumoniae*	3	23/12/2005	E2	ND	220, 60 ^f^	*aac(6’)Ib-cr*	ND	ND	ND	ND	ND	ND	ND	ND	ND	ND	ND
6728	*K. pneumoniae*	3	17/03/2007	E3	ND	220, 60 ^f^	*aac(6’)Ib-cr*	ND	ND	ND	ND	ND	ND	ND	ND	ND	ND	ND
6736	*K. pneumoniae*	3	23/07/2007	E3	+	60	*aac(6’)Ib-cr*	-	-	-	SHV-12^g^,^h^	4	0.25	4	128	> 256	4	8
6722	*K. pneumoniae*	3	12/06/2005	E4	ND	220, 60 ^f^	*aac(6’)Ib-cr*	ND	ND	ND	ND	ND	ND	ND	ND	ND	ND	ND
6737	*K. pneumoniae*	3	25/03/2007	E4	+	220, 60	*aac(6’)Ib-cr*	-	-	-	SHV-12^g^,^h^	4	0.25	4	128	> 256	4	4
6738	*K. pneumoniae*	3	25/11/2007	E5	ND	60 ^f^	*aac(6’)Ib-cr*	ND	ND	ND	ND	ND	ND	ND	ND	ND	ND	ND
6739	*K. pneumoniae*	3	26/11/2007	E5	ND	60 ^f^	*aac(6’)Ib-cr*	ND	ND	ND	ND	ND	ND	ND	ND	ND	ND	ND
6733	*K. pneumoniae*	3	22/03/2007	E6	+	220, 60	*aac(6’)Ib-cr*	-	-	-	SHV-12^g^,^h^	4	0.25	4	128	> 256	8	4
6727	*K. pneumoniae*	3	15/03/2007	E7	ND	220 ^f^	*aac(6’)Ib-cr*	ND	ND	ND	ND	ND	ND	ND	ND	ND	ND	ND
6735	*K. pneumoniae*	3	23/03/2007	E8	+	60	*aac(6’)Ib-cr*	-	-	-	SHV-12^g^,^h^	4	0.25	2	256	> 256	4	8
6729	*K. pneumoniae*	3	19/02/2007	E9	ND	220 ^f^	*aac(6’)Ib-cr*	ND	ND	ND	ND	ND	ND	ND	ND	ND	ND	ND
01-1600	*K. pneumoniae*	1	11/04/2010	NR	-	180	*qnrA1*	Leu	-	Iso	CTX-M.15^h^	> 256	> 64	> 64	> 256	> 256	4	2
01-1634	*K. pneumoniae*	1	02/09/2010	NR	-	130, 80	*qnrB1*	Iso	-	Iso	CTX-M-15^h^	> 256	> 64	> 64	> 256	128	4	2
06-1605	*K. pneumoniae*	4	17/02/2011	NR	+	180	*qnrB5*	Leu	-	Iso	CTX-M-15^h^	> 256	> 64	16	> 256	> 256	4	> 64
839	*K. pneumoniae*	4	17/03/2011	NR	+	280	*qnrB1*	-	-	-	ND^f^,^g^	32	1	4	> 256	> 256	32	> 64
837	*K. pneumoniae*	4	24/03/2011	NR	+	230	*qnrB1*	-	-	-	CTX-M-15^h^	8	2	4	> 256	32	4	> 64

Hospitals: 1. Hospital Civil de Guadalajara (HCG); 2. Hospital de Pediatría CMN Siglo XXI (HPCMN); 3. Hospital General de Tapachula Chiapas (HGTC); 4. Hospital del Niño de Tabasco (HN); 5. Hospital Universitario (HU).

b The XbaI restriction profiles showed different DNA patterns among the clinical isolates of their respective species. The number in parenthesis corresponds to the number of strains with the same PFGE pattern.

c The underlined plasmids correspond to conjugative plasmids.

d wild-type gene. Amino acids; Ser, serine; Asp, aspartic acid; Leu, leucine; Asn, asparagine.

e In this hospital 17 pediatric isolates were analyzed; 13 corresponded to the same clone (E) and four were not related isolates.

f The plasmid profile correspond to clinical isolate, due to the mating experiment was not assayed.

g β-lactamase TEM-1 was identified by PCR and sequencing.

h β-lactamase SHV-11 was identified by PCR and sequencing.

NR, not related; NA, not analyzed; ND, not determined.

In terms of the protein mutation percentages, the analysis revealed the following: 37.5% and 25% concerning the GyrA and ParC proteins, respectively, from the *E. coli* isolates, and 12.5% with respect to the same proteins from the *K. pneumoniae* isolates. The GyrA protein in the *E. cloacae* isolates corresponded to a wild type, and the *parC* gene from the *E. cloacae* isolates was not analyzed. In general, the most frequently identified mutations were the Ser83Leu for GyrA, in 85.7% (6/7) of the isolates, and the Ser80Iso for ParC, in 100% of them (Table 2 and 3).

**Figure 2:**
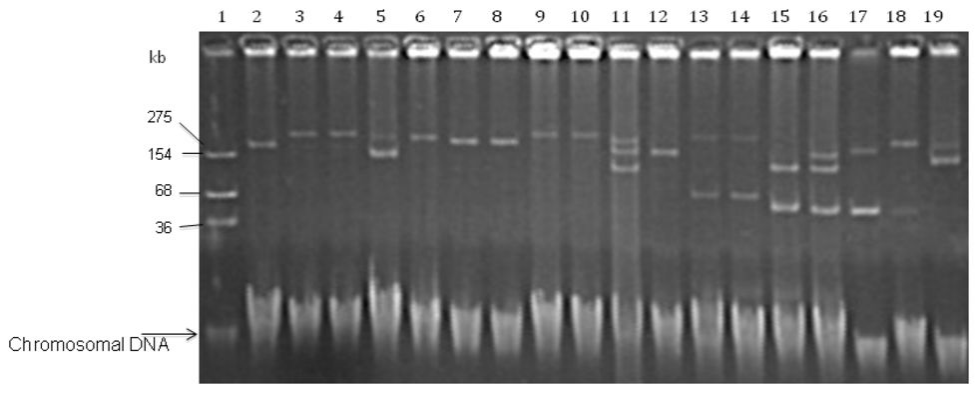
Plasmid profile of representative clinical isolates and transconjugants. 1. *E. coli* 50192 (154-, 68-, and 36-kb), 2. pUA21 (275 kb), 3. 835, 4. T835, 5. 836, 6. T836, 7. 837, 8. T837, 9. 839, 10. T839, 11. 840, 12. T840, 13. 843, 14. T843, 15. T850, 16. 850, 17. 6737, 18. T6737, 19. 844.

Plasmid DNA was extracted from all the *qnr*-carrying isolates. All isolates contained from one to five plasmids, within a size range of 60- to 320-kb. As for the mating experiments, nine out of 17 (52.9%) were successful (Table 2 and Figure 2). The *qnrB1-19-6-5* alleles were the *qnr*-determinants co-transferred with ESBL CTX-M-15, whereas the *aac(6*’)*-Ib-cr-*positive isolate turned out to be negative in terms of the ESBL SHV-12 genes screened for (Table 4). Incompatibility group FII was identified in all *qnr*-positive transconjugants and their parental isolate; however, two isolates (T837 and T843) tested positive for incompatibility groups FIB and repF, as well as for FIIB and L/M, respectively (Table 4). Five of the transconjugants showed a two to three-fold increase in the MIC for nalidixic acid in comparison to *E. coli* J53.

**Table 4:** Genetic characteristics of the transconjugants that acquired PMQR genes.

Transconjugant number	Plasmid(s) (kb)	PMQR genes	ESBL-type	Plasmid incompatibility group (Inc)				MIC	(mg/L)		
					NAL	CPO	LEV	CTX	CAZ	CAZ/CLV	Gm
T835	300	*qnrB1*	Neg^b^	FIIs	16	0.0625	0.125	128	> 256	4	> 64
T836	250	*qnrB1*	CTX-M-15	FIIs	16	0.0625	0.125	> 256	128	4	2
T837	230	*qnrB1*	CTX-M15	FIIs, FIB	8	0.0625	0.0625	> 256	32	4	> 64
T839	280	*qnrB1*	Neg^b^	FIIs	4	0.002	0.004	64	>256	4	> 64
T840	200	*qnrB19*	CTXM-15	FIIs	8	0.0625	0.125	> 256	32	4	> 64
T843	260, 90	*qnrB6*	CTXM-15	FIIs, Frep, FIB, L/M	4	0.032	0.015	> 256	128	4	4
T850	140, 60	*qnrB19*	Neg^b^	FIIs	16	0.0625	0.25	32	128	64	4
T06-1605	180	*qnrB5*	CTX-M15	FIIs	2	0.002	0.004	1	4	4	0.5
T6737	250, 60	*aac(6’)-Ib-cr^a^*	Neg^b^	FIIs	4	0.03	0.008	128	128	4	2

^a^ The *aac(6*’)*-Ib-cr* was identified by PCR digestion with enzyme BstI5.

^b^ β-lactamase TEM-1 was identified by PCR.

Neg, negative.

Characteristics of *aac(6*’)*-Ib-cr* and *qepA*-positive isolates

The *aac(6*’)*-Ib-cr*-positive *K. pneumoniae* isolates corresponded to clone E (thirteen related isolates, E1 to E9, and four unrelated ones, NR) (Table 2, Figure 1). Some isolate from E clone and all from the NR isolates were characterized, all isolates displayed a phenotype susceptible to quinolone and fluoroquinolone antibiotics and all of them bore the wild-type *gyrA* and *parC* genes. Furthermore, all isolates contained ESBL SHV-12, which provided a cephalosporin-resistant phenotype (Table 2). In general, the *aac(6*’)*-Ib-cr*-positive isolates from clone E and NR showed an heterogeneous plasmid pattern, however in the clone E at least a 220- or 60-kb plasmid were identified in majority of isolates (Table 3). In transconjugant T6737, obtained by mating one of the isolate from clone E (use as a representative isolate), the *aac(6*’)*-Ib-cr* gene was transferred onto a 220-kb plasmid corresponding to incompatibility group FIIs. This transconjugant displayed a different phenotype than the parental isolate; however, it displayed a three-fold increase in the MIC for ciprofloxacin compared to *E. coli* J53 (Table 4).

The two-*qepA* genes corresponded to the *qepA1* allele. This gene was identified in two *qnr*-negative *E. coli* isolates from the same hospital (Hospital 2, HPCMN) (Table 1), corresponding to clone B, obtained in 2009 (Table 2 and 3). These *E. coli* isolates showed a multidrug resistance pattern, except to gentamicin; they harbored ESBL CTX-M-15, and had a plasmid pattern consisting of 180- and 120-kb plasmids. However, no transconjugants were obtained. Mutations Ser-83-Leu and Asp-87-Asn with respect to the GyrA protein and Ser-80-Iso with respect to the ParC protein were identified (Table 2). 

## Discussion

Unlike β-lactam antibiotics, quinolone antimicrobial agents were not isolated from living organisms, but rather they were synthesized by chemists. This group of antibiotics was developed in the late 1960s starting with the accidental discovery of nalidixic acid during the synthesis of chloroquine, an antimalarial compound. These antibiotics have been used in human adult patients to treat urinary tract infections, and the development of quinolones has resulted in the expansion of their clinical applications to include the treatment of infections at many body sites. However, the routine use of nalidixic acid and fluoroquinolones in pediatric patients must be evaluated, due to the risk of hepatotoxicity [23,24]. Nevertheless, an increase in the prescription of quinolone and fluoroquinolone antibiotic agents for the treatment of bacterial infections both in hospital settings and in the community has taken place in recent years [9]. Historically, chromosomal mutations have occurred in the *gyrA* and the *gyrB* genes, which are responsible for coding the A and B subunits of DNA gyrase, and in the *parC* and the *parE* genes, which are responsible for coding the C and E subunits of topoisomerase IV; these have been the main molecular mechanisms that confer resistance to this class of antibiotics. In fact, three plasmid-mediated quinolone resistance genes --*qnr-, aac(6*’)*-Ib-cr*, and *qepA*-- that confer a low-level resistance to quinolones and fluoroquinolones through different mechanisms have been described. The function of the Qnr proteins that confer quinolone resistance is to protect the gyrase proteins, the AAC(6’)-Ib-cr protein, is an acetyltransferase that modifies the quinolones, and the QepA protein is a active efflux pump [10]. 

 Despite the fact that these genes are not capable of conferring resistance against the most clinically important quinolones, these enzymes are a great cause of concern because they promote the selection of chromosomal mutations (the above-mentioned GyrA and ParC mutations) [25]. In addition, they are often associated to different ESBL families [8]. For these reasons, we had previously characterized the PMQR genes in ESBL-producing *Enterobacteriaceae* clinical isolates causing nosocomial infection in adults in Mexican hospitals [12]. The present work describes the characterization of PMQR genes in ESBL-producing *Enterobacteriaceae* clinical isolates causing nosocomial infection in the pediatric population from several Mexican hospitals.

Both studies included only ESBL-producing clinical isolates, and the cephalosporin- and quinolone-resistant genes were the ones characterized. Clear differences between pediatric and adult isolates were identified. In the adult isolates, the ESBLs SHV-type was the most prevalent one (81.6%), followed by ESBL CTX-M-15 (44.9%) [12]. Nevertheless, in the *qnr*-positive pediatric isolates, CTX-M-15 was the most prevalent ESBL identified (70.5%). In addition, it is noteworthy that in the pediatric isolates neither of the ESBL genes was encoded in combination with another ESBL, in contrast with the adult isolates. However, in both bacterial populations, the combination of ESBL and PMQR genes may be pointing to a co-selection of cephalosporin and quinolone resistance. On the other hand, this characteristic could be related to the few clonal groups identified among the PMQR gene-positive pediatric isolates, in comparison with the adult isolates among which some clones have persisted over time in a few hospitals.

With respect to the PMQR genes in both populations, the prevalence in pediatric isolates was lower for the *qnr*- and *aac(6*’)*-Ib-cr* determinants (15.1%), but the same for the *qepA1* gene (1.7%). In general, the prevalence of *qnr*-determinants was higher among the *E. cloacae* isolates (23.5% and 55.8%), followed by the *K. pneumoniae* (13.7% and 50%) and the *E. coli* isolates (13.6% and 1.4%) in both works. However, the prevalence in *E. coli* isolates was higher in the pediatric patients. Few reports have assessed the PMQR genes in ESBL-producing *Enterobacteriaceae* pediatric isolates [26,27]. Nevertheless, a study of ESBL- or AmpC-producing *E. coli* clinical isolates study was carried out in pediatric patients in China; prevalence of PMQR genes was determined to be 6.8%, of which 4.1% corresponded to *qnr* alleles (*qnrA*, *B* and *S*), 3.4% to the *aac(6*’)*-Ib-cr* gene, and the *qepA* gene was not identified [26]. Results were similar in a study carried out in Uruguay: out of the 5.4% of ESBL-producing *Enterobacteriaceae* pediatric isolates, 20% (5/20) tested positive for certain PMQR genes; four isolates corresponded to *qnr* alleles, and one isolate, to the *aac(6*’)*-Ib-cr* gene [27].

Among the PMQR-genes identified in this study, the *qnrB-*alleles and the *aac(6*’)*-Ib-cr* gene were the most prevalent, whereas the *qnrS1, qnrA1* and *qnrB*-like alleles were the most prevalent in China and Uruguay. With respect to the rate of the *aac(6*’)*-Ib-cr* gene, 3.4% was identified in China, 5% in Uruguay, and 15.1% in Mexico. In this study, the prevalence of the *qepA1* gene among pediatrics isolates was low (1.7%), as has been generally reported; the gene was not identified in China and it was not screened in Uruguay [26,27]. Among both pediatric and adult isolates in Mexico, all *qepA1*-positive isolates corresponded to *qnr*-negative *E. coli*. They were identified mainly in combination with ESBL CTX-M-15; and they were co-transferred with CTX-M-15 in one adult isolate [12]. As for QRDR mutations in the *gyrA* and *parC* genes in PMQR gene-positive adult isolates, the prevalence was higher (69.3% and 80%, respectively), compared to the pediatric isolates (41.1% and 35.2%, respectively). However, the chromosomal genes for type II topoisomerases (GyrA and ParC proteins) were not analyzed in isolates in China or Uruguay.

The acquisition of multidrug-resistance due to horizontal transmission events involving several plasmids with different incompatibility groups has been documented [28]. In this work, the *qnrB1* alleles and the *aac(6*’)*-Ib-cr* genes on transferable plasmids were identified as corresponding to one main incompatibility group (FIIs), in contrast with the conjugative plasmids identified in the adult isolates (IncF_rep_ and IncN). However, the incompatibility groups identified in the ESBL-producing *Enterobacteriaceae* pediatric isolates in Uruguay was heterogeneous. Such cotransmissibility of PMQR and ESBL genes could correspond to the acquisition of different genetic elements in both adult and pediatric isolates.

In conclusion, the characterization of PMQR and ESBL genes on pediatric isolates from ESBL-producing *Enterobacteriaceae* showed marked differences with respect to the adult ones: i) In general the prevalence of PMQR genes (*qnr* and *aac(6*’)*-Ib-cr*) was much lower in the pediatric isolates, compared to the adult isolates in Mexico; ii) the *aac(6*’)*-Ib-cr* gene that confers ciprofloxacin resistance was mainly identified in the *K. pneumoniae* pediatric isolates, whereas in *E. coli* were mainly identified in adult isolates iii) the mutations in gyrase and topoisomerase IV identified on the *qnr*-positive isolates were fewer in the pediatric isolates compared to the adult ones; iv) ESBL CTX-M-15 was the main ESBL identified in the pediatric isolates, whereas in the adult ones ESBLs corresponded to the CTX-M and the SHV families; v) the prevalence of PMQR genes in the pediatric isolates was higher in comparison to the results from China, and it was similar to those from Uruguay, another Latin American country. These points highlight the need to use antibiotics such as cephalosporins and quinolones with caution when treating pediatric nosocomial infections.
